# Enhanced Magnetocaloric Properties of the (MnNi)_0.6_Si_0.62_(FeCo)_0.4_Ge_0.38_ High-Entropy Alloy Obtained by Co Substitution

**DOI:** 10.3390/e26090799

**Published:** 2024-09-19

**Authors:** Zhigang Zheng, Pengyan Huang, Xinglin Chen, Hongyu Wang, Shan Da, Gang Wang, Zhaoguo Qiu, Dechang Zeng

**Affiliations:** 1 School of Materials Science & Engineering, South China University of Technology, Guangzhou 510640, China; mspyhuang@mail.scut.edu.cn (P.H.); msxlchen@mail.scut.edu.cn (X.C.); mshywang@mail.scut.edu.cn (H.W.); mssda@mail.scut.edu.cn (S.D.); msgwang@scut.edu.cn (G.W.); zgqiu@scut.edu.cn (Z.Q.); medczeng@scut.edu.cn (D.Z.); 2 Yangjiang Branch, Guangdong Laboratory Materials Science and Technology Yangjing Advanced Alloys Laboratory, Yangjiang 529599, China

**Keywords:** magnetocaloric effect, high-entropy alloys, component optimization, configurational entropy

## Abstract

In order to improve the magnetocaloric properties of MnNiSi-based alloys, a new type of high-entropy magnetocaloric alloy was constructed. In this work, Mn_0.6_Ni_1−_*_x_*Si_0.62_Fe_0.4_Co*_x_*Ge_0.38_ (*x* = 0.4, 0.45, and 0.5) are found to exhibit magnetostructural first-order phase transitions from high-temperature Ni_2_In-type phases to low-temperature TiNiSi-type phases so that the alloys can achieve giant magnetocaloric effects. We investigate why *c_hexagonal_*/*a_hexagonal_* (*c_hexa_*/*a_hexa_*) gradually increases upon Co substitution, while phase transition temperature (*T_tr_*) and isothermal magnetic entropy change (Δ*S_M_*) tend to gradually decrease. In particular, the *x* = 0.4 alloy with remarkable magnetocaloric properties is obtained by tuning Co/Ni, which shows a giant entropy change of 48.5 J∙kg^−1^K^−1^ at 309 K for 5 T and an adiabatic temperature change (Δ*T_ad_*) of 8.6 K at 306.5 K. Moreover, the *x* = 0.55 HEA shows great hardness and compressive strength with values of 552 HV2 and 267 MPa, respectively, indicating that the mechanical properties undergo an effective enhancement. The large Δ*S_M_* and Δ*T_ad_* may enable the MnNiSi-based HEAs to become a potential commercialized magnetocaloric material.

## 1. Introduction

Solid-state refrigeration technology is a new type of refrigeration technology with the advantages of environmental protection, energy savings, high efficiency, stability, and reliability [1,2,3,4]. It is feasibly regarded as the next generation of refrigeration technology by the United States Department of Energy. Solid-state refrigeration technologies are mainly divided into magnetic refrigeration technology [5], thermoelectric refrigeration technology [6,7], and mechanical thermal refrigeration technology [8], including multicaloric refrigeration by multiple fields (magnetic fields, pressure fields, electric fields, etc.) [9,10,11]. The magnetocaloric effect (MCE) seen when using magnetic materials as the working medium, exhibiting very low levels of energy consumption, is the basic principle of magnetic refrigeration [12,13]. The working efficiency of magnetic refrigeration can reach 30–60% of the Carnot cycle, which is much higher than that of the vapor compression refrigeration and several other solid-state refrigeration technologies. Hence, magnetic refrigeration is widely considered to be the next generation of refrigeration technology with a wide operating temperature range, stable operation without noise, and long service life.

MnNiSi-based magnetic refrigeration materials have attracted more and more attention from researchers because of the large magnetocaloric effect and wide adjustable Curie temperature window (CTW) effect. These alloys families exhibit a martensitic-like phase transition from Ni_2_In-type structure to TiNiSi-type structure, and the transformation process is accompanied by giant negative thermal expansion. However, the poor mechanical properties induced by a severe first-order phase transition (FOPT) hinder the commercialization of MnNiSi-based alloys. For example, the (MnNiSi)_0.62_(FeCoGe)_0.38_ [14] HEA, which struggles to maintain its integrity due to poor mechanical properties, shows giant caloric effect under magnetic field and pressure field. Giant changes in crystal structure and internal stress of these types of alloys induced by drastic first-order phase transformation will cause the alloys to tend to crack and lead to the deterioration of its mechanical properties. Several researchers have worked extensively to solve this problem. F. Zhu et al. [15] improved the mechanical properties of Mn_0.98_CoGe alloy by epoxy bonding. They found that the compression strength of the alloy reached 152 MPa when the epoxy resin content was 3 wt.% and 218 MPa when it was 5 wt.%. H. Zhou et al. [16] prepared low-melting metal-bonded MM’X/In composites to improve mechanical properties. The compressive strength of the alloy with 25 wt.% In reached 48 MPa. When the In content was 30 wt.%, the thermal conductivity of the alloy was enhanced by more than 8 times compared to the samples bonded with epoxy resin. They believe this results from the low-porosity compact structure formed by the high ductility of In, which greatly improved the mechanical properties and thermal conductivity of the alloy.

Recently, more and more researchers have paid attention to high-entropy alloys (HEAs) due to their outstanding mechanical properties [17,18], corrosion resistance [19,20], radiation resistance [21,22], and low-temperature performance [23]. There are generally two definitions of HEAs [24]. From the perspective of mixing and phase formation, the mixing entropy of high-entropy alloy systems is very high, which is composed of configurational entropy, magnetic entropy, vibrational entropy, and electronic entropy. Out of these, configurational entropy is the most important factor. Therefore, alloys can be distinguished into low-entropy alloys (${∆S}_{conf}<1R$), medium-entropy alloy ($1R\;\leq {∆S}_{conf}\leq 1.5R$), and high-entropy alloys (${∆S}_{conf}>1.5R$) according to the configuration entropy. The calculation formula of configuration entropy is shown in Equation (1):

(1) $${∆S}_{conf}=-R\sum_{i=1}^{n}c_{i}\ln c_{i}$$

where *R* is a gas constant with a value of 8.314 J·K^−1^ mol, *n* is the total number of elements, and $c_{i}$ is the mole fraction of the *i*th component.

Meanwhile, HEAs are defined as alloys with five or more elements. Each major element is of 5–35 at%, while the content of each minor element is less than 5 at%. As an important direction of functional HEAs, the published works on magnetocaloric high-entropy alloys (MCE-HEAs) are less than 0.5% of the total HEA research, while most MCE-HEAs exhibiting giant magnetocaloric effects include rare-earth-based elements [25,26]. It was reported that (FeMnNi)_66.7_(Ge_0.45_Si_0.55_)_33.3_ [27] exhibited a giant isothermal entropy change of 13 J∙kg^−1^K^−1^ at 2.5 T, which was comparable to the notable MCE-HEAs Gd_20_Dy_20_Er_20_Ho_20_Tb_20_ [28] (isothermal entropy change of8.6 J∙kg^−1^K^−1^ at 5 T). More MCE-HEAs of rare-earth-free (RE-free) show surprising magnetocaloric effects, which indicated that RE-free HEAs have great research potential when applied for refrigeration. However, it is a problem that great magnetocaloric effects are generally accompanied by limited mechanical properties in MM’X magnetocaloric materials. An ideal combination of great MCE and mechanical properties would be achieved by designing the magnetocaloric materials as high-entropy alloys. Therefore, it is worthwhile exploring the potential application of new magnetocaloric high-entropy alloys.

We previously discussed the effect of the Si/Ge ratio on MM’X alloys in the published article [29]. On this basis, we selected the sample with the best performance to further study the effect of Ni/Co ratio on the magnetic and mechanical properties of MM’X alloys. The phase transition temperature of alloys may also be adjusted by tuning the content of Ni, which makes it possible to achieve a combination of magnetocaloric and mechanical properties.

In this work, we aim to prepare Mn_0.6_Ni_1−*x*_Si_0.62_Fe_0.4_Co*_x_*Ge_0.38_ (*x* = 0.4, 0.45, 0.5, and 0.55) alloys by tuning the Ni/Co ratio; these exhibit a phase transformation from Ni_2_In-type structures at high temperatures to TiNiSi-type structures at low temperatures. Thus, the magnetocaloric, microstructural, and the mechanical properties observed with the variation in the Ni/Co ratio are studied, demonstrating that the compounds exhibit large MCE.

## 2. Experimental

**Figure 1:**
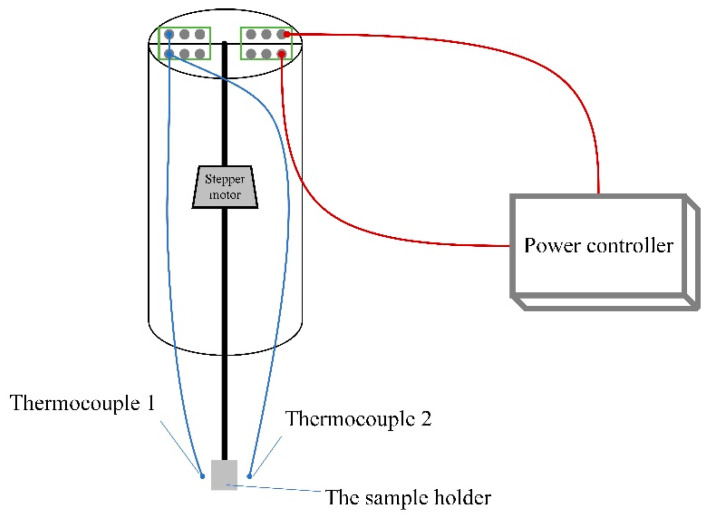
The schematic diagram of the PPMS-based adiabatic temperature change direct measurement device.

The HEAs with different contents of Co-doping were prepared by the arc-melting method using elements with purity higher than 99.99 wt.% under argon atmosphere. An excess of 5 at% of Mn was added due to the volatility of Mn at low-saturation vapor pressure. The as-cast ingots were annealed in high-vacuum quartz tubes at 750 °C for 72 h and then quenched in water to avoid residual stress. The crystal structure at room temperature of four HEAs was characterized by X-ray diffraction (XRD, X’pert powder, PANalytical, Almelo, The Netherlands) using Cu Kα radiation. The Rietveld refinement method based on the XRD patterns was carried out to identify the crystal structure and lattice parameters using Rietica v4.2 software. The martensitic transformation temperature was analyzed by a differential scanning calorimeter (NETZSCH DSC 214, Selb, Germany) with a ramp rate of 10 K∙min^−1^ under nitrogen atmosphere. Magnetic measurements were obtained using a vibrating sample magnetometer (VSM, Quantum Design, San Diego, CA, USA) equipped in a physical properties measurement system (PPMS). The measurement of adiabatic temperature change (Δ*T_ad_*) was obtained in a self-developed PPMS-based adiabatic temperature change direct measurement device, and the schematic diagram is shown in Figure 1. The residence time of the sample in the magnetic field was 120 s, the entry and exit rate of the sample rod was 180 mm/s, and the heating rate in the sample chamber was 0.5 K∙min^−1^. Mechanical properties were measured using a small-load Vickers hardness tester (HVS-10, Innova test, Amsterdam, The Netherlands) with a 2000 g hold for 10 s and universal testing machine (AG-X 100 KN, Shimadzu, Kyoto, Japan). The element distribution was characterized by energy-dispersive spectroscopy (EDS) equipped in a scanning electron microscope (SEM, Carl Zeiss AG, Oberkochen, Germany).

## 3. Results and Discussion

**Figure 2:**
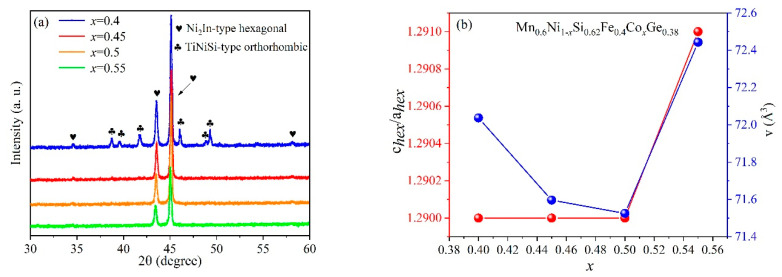
(**a**) The X-ray diffraction patterns of HEAs with different Co-doping at 295 K. (**b**) Unit cell parameters *c_hex_/a_hex_* and volume *v* for Mn_0.6_Ni_1−*x*_Si_0.62_Fe_0.4_Co*_x_*Ge_0.38_ (*x* = 0.4, 0.45, 0.5, 0.55) alloys determined from Rietveld refinements.

The Mn_0.6_Ni_1−*x*_Si_0.62_Fe_0.4_Co*_x_*Ge_0.38_ (*x* = 0.4, 0.45, 0.5, and 0.55) alloys for X-ray diffraction obtained by heat treatment were ground into fine powder. The results of alloys in terms of powder pattern with different contents of Co-doping at room temperature are shown in Figure 2a. With the decrease in *x*, Mn_0.6_Ni_1−*x*_Si_0.62_Fe_0.4_Co*_x_*Ge_0.38_ (*x* = 0.4, 0.45, 0.5, and 0.55) alloys transform from a single high-temperature hexagonal Ni_2_In-type phase (space group P63/mmc) to a biphasic coexistence of hexagonal Ni_2_In-type and orthorhombic TiNiSi-type (space group Pnam).

In order to investigate the crystal structure and the content of different phases at 295 K, the Rietveld method was used to carry out full-spectrum fitting lattice refinement (shown in Figure 2a). The *c_hex_/a_hex_* and cell volume *v* for HEAs with different Co-doping levels are shown in Figure 2b. Table 1 lists all structural refinement data. The *x* = 0.4 HEA contains 98.6% hexagonal Ni_2_In-type phase and 1.4% orthorhombic TiNiSi-type phase. Another three alloys exhibit 100% hexagonal Ni_2_In-type phase. The MM’X system alloys undergo the structural transition from hexagonal Ni_2_In-type phase at high temperatures to orthogonal TiNiSi-type phase at low temperatures. The corresponding lattice constants of hexagonal and orthorhombic structure are shown as follows [29,30]:

(2) $$a_{\mathrm{orth}}=c_{\mathrm{hex}},\;b_{\mathrm{orth}}=a_{\mathrm{hex}},\;c_{\mathrm{orth}}=\sqrt{3}a_{\mathrm{hex}},\;v_{\mathrm{orth}}=2v_{\mathrm{hex}}$$

The *c_hex_*/*a_hex_* and *a_orth_*/*b_orth_* are closely related to the stability of the crystal structure. The smaller the values, the more stable the corresponding phase structure [31].The negative thermal expansion rate of the *x* = 0.4 HEA can be calculated using Equation (2) [32]. From the hexagonal structure to the orthorhombic structure, the crystal volume of the alloy shows a negative thermal expansion of 5.51%. Compared to this alloy system, such as Mn_0.94_Fe_0.06_NiGe [32], *x* = 0.4 HEA shows an outstanding negative thermal expansion. Figure 2b clearly shows that the *c_hex_*/*a_hex_* gradually increases with the increasing Co-doping. The cell volume of hexagonal phase decreases and then increases with Co-doping. XRD cannot clearly distinguish the proportion of Ni and Co atoms at the same atomic sites. Our previous study [29] and other published references [33,34] have reported the atomic occupancy of MM’X (M, M’ = transition metals, X = carbon or boron group elements) alloys, which exhibited a phase transition from Ni_2_In-type structures to TiNiSi-type structures. The M element with a fewer number of valence electrons tends to occupy the (0, 0, 0) and (0, 0, 0.5) sites, while the M’ element with a greater number of valence electrons is inclined to occupy the (1/3, 2/3, 3/4) and (2/3, 1/3, 1/4). The X element occupies the (1/3, 2/3, 1/4) and (2/3, 1/3, 3/4) sites. All elements are homogenously distributed in the samples without component segregation (as shown in Supplementary Figure S1).

Δ*v*/*v* = (*v_orth_*/2 − *v_hex_*)/*v_hex_* (3)

**Figure 3:**
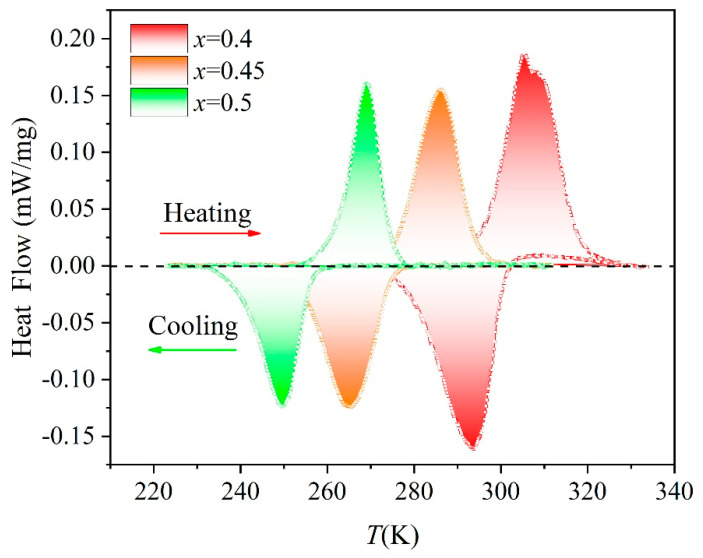
DSC curves during heating process around *T_C_* for HEAs with different Co-doping.

Figure 3 shows the heat flow curves of the HEAs with different Co-doping arrangements are near the Curie temperature (*T_C_*). It is observed that endothermic peaks appear near the *T_C_* of alloys *x* = 0.4, 0.45, and 0.5, and the corresponding peaks are 306 K, 286 K, and 269 K, respectively. However, neither endothermic peaks nor exothermic peaks observed in Figure 3 appear in alloys *x* = 0.55, which is due to the fact that *T_tr_* is outside the operational range (183–400 K) of DSC. It is suggested that Co substitution stabilizes the Ni_2_In-type phase, which shifts the phase temperature of structure to a lower temperature. The decreasing structural transition temperature with the substitution of Co for Ni is attributed to the strengthening covalent bonding between neighboring Mn-Mn atoms [34,35,36]. Therefore, it may be a sufficient method to tailor the *T_tr_* to the Curie temperature window and then achieve the magnetostructural transition. The DSC heat flow curves of *x* = 0.4, 0.45, and 0.5 alloys show that the transition temperature *T_tr_* is lower than the Curie temperature *T_C_*. Thus, the magnetic and structural coupling of the three alloys occurs, resulting in a giant magnetocaloric effect.

(4) $$L=\int_{T_{s}}^{T_{f}}\frac{dQ}{dT}dT,∆S=L/T_{C}$$

Equation (4) can be used to calculate the latent heat of DSC heat flow curve phase transition and DSC entropy changes ($-∆S_{DSC}^{peak}$) of alloys, which are 56.4 J∙kg^−1^K^−1^, 27.3 J∙kg^−1^K^−1^, and 24.8 J∙kg^−1^K^−1^ for *x* = 0.4, 0.45, and 0.5, respectively.

**Figure 4:**
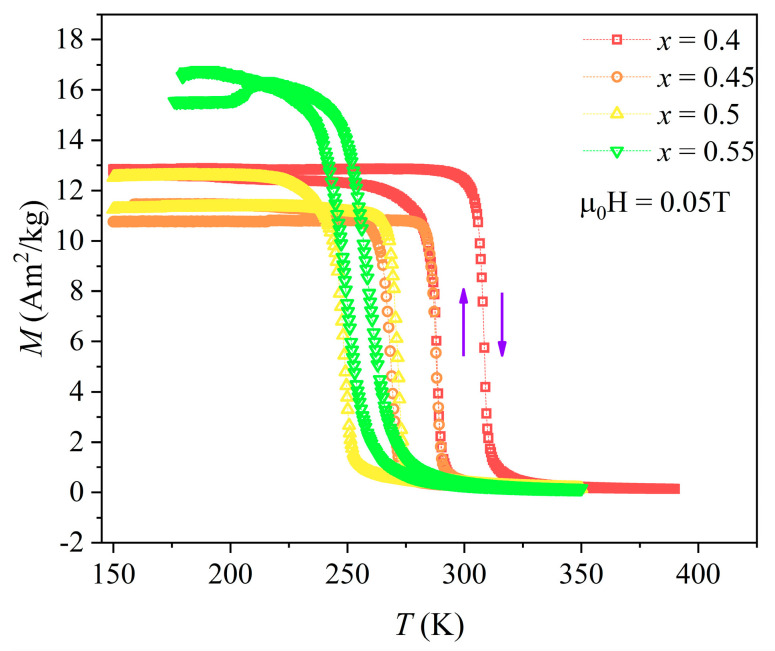
Thermomagnetic curves of the HEAs with different Co-doping during the heating and cooling process at 0.05 T.

Figure 4 shows the temperature-dependent magnetization curves of Mn_0.6_Ni_1−*x*_Si_0.62_Fe_0.4_Co*_x_*Ge_0.38_ (*x* = 0.4, 0.45, 0.5, 0.55) alloys at a magnetic field of 0.05 T. The curves of all alloys in Figure 4 are smooth. For *x* = 0.40–0.50, the thermomagnetic results show the magnetostructural transformation between ferromagnetic and paramagnetic phases. The *T_C_* was obtained as the maximum of the derivative of the thermomagnetic curves during the heating process and was 309 K, 289 K, 272 K, and 259 K. It is clear that *T_C_* gradually decreases with the substitution of Co for Ni. The discrepancy between *T_C_* in cooling and heating processes is also observed, namely there is thermal hysteresis (Δ*T_hys_*). By calculation, the Δ*T_hys_* of *x* = 0.4, 0.45, 0.5, and 0.55 alloys are 21 K, 20 K, 24 K, and 12 K, respectively. Therefore, *T_C_* gradually decreases from near room temperature to below room temperature as the Co content increases.

**Figure 5:**
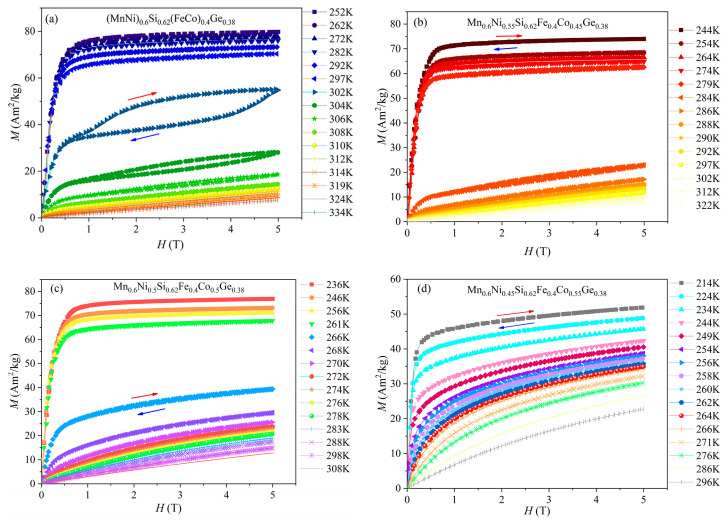
Isothermal magnetization and demagnetization curves around *T_C_* for (**a**) *x* = 0.4, (**b**) *x* = 0.45, (**c**) *x* = 0.5, (**d**) *x* = 0.55. The red arrows indicate magnetization, and the blue arrows indicate demagnetization.

Figure 5a–d show the isothermal magnetization curves of HEAs, with different Co-doping arrangements, near the Curie temperature. All alloys exhibit obvious magnetic transformation with rising temperatures. When the temperature is lower than *T_C_*, the isothermal magnetization curves of alloys are ferromagnetic, and the magnetization increases rapidly with the increasing intensity of the magnetic field. The curves turn rapidly at about 1T, and no longer increase when approaching saturation. When the temperature is higher than *T_C_*, the isothermal magnetization curves of the alloys show a nearly linear change, which is characteristic of paramagnetic states. The *x* = 0.4 alloy undergoing first-order phase transition was observed to exhibit a giant magnetocaloric effect in a very limited temperature range, which was accompanied by an obvious magnetic hysteresis. The curves with magnetic hysteresis of *x* = 0.45 (*x* = 0.5) are obtained in the temperature range near-phase transition from 279 K to 284 K (261 K to 266 K), which are not shown in Figure 5. For *x* = 0.55 alloys, the initial magnetization curve and demagnetization were almost identical, indicating that the magnetic hysteresis is almost negligible. This sample undergoes second-order phase transition, which does not show any hysteresis.

Isothermal magnetic entropy (Δ*S_M_*) is a significant method used to evaluate the magnetocaloric properties of magnetocaloric materials, which can be obtained from Figure 5. According to the well-known Maxwell relation, the calculation formula of Δ*S_M_* is as follows:

(5) $$∆S_{M}\left(T,H\right)=-\int_{0}^{H}\left(\frac{\partial M}{\partial T}\right)dH$$

**Figure 6:**
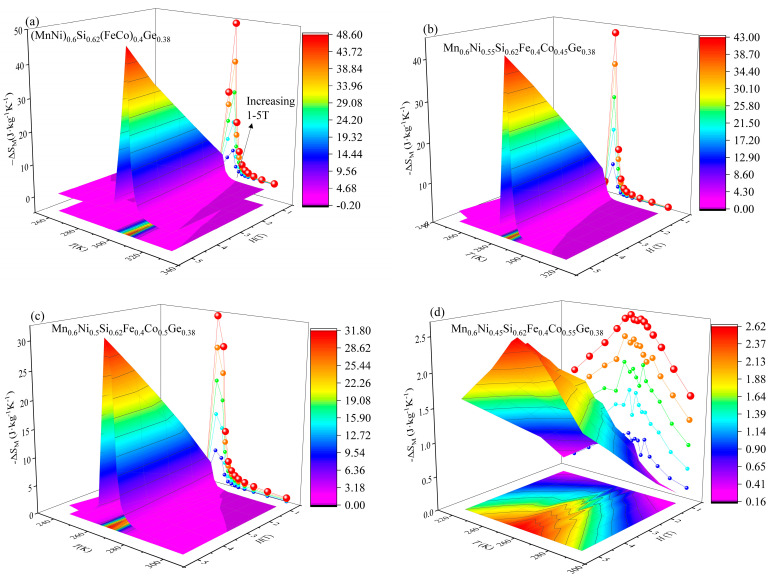
Three—dimensional surfaces showing −Δ*S_M_* of (**a**) *x* = 0.4, (**b**) *x* = 0.45, (**c**) *x* = 0.5, (**d**) *x* = 0.55 under Δ*H* from 1 T to 5 T. The plots with the contour map in the plane of −Δ*S_M_* are projected from 3D surfaces.

Figure 6 shows the 3D diagram of Δ*S_M_* near the Curie temperature of Mn_0.6_Ni_1−*x*_Si_0.62_Fe_0.4_Co*_x_*Ge_0.38_ with *x* = 0.4, 0.45, and 0.5 alloys at a magnetic field of 0–5 T. As shown in Figure 6a–d, when the magnetic field intensity reaches 5 T, the maximum isothermal magnetic entropy changes ${∆S}_{M}^{peak}$ of *x* = 0.4, 0.45, 0.5, and 0.55 are 48.5 J∙kg^−1^K^−1^, 42.9 J∙kg^−1^K^−1^, 31.7 J∙kg^−1^K^−1^, and 2.6 J∙kg^−1^K^−1^, respectively. The *x* = 0.4, 0.45, and 0.5 alloys have a giant Δ*S_M_*, showing the characteristics of FOPT. The alloy *x* = 0.55 has a small isothermal magnetic entropy change, which is characteristic of second-order phase transition (SOPT) materials. The mechanism of the phase transition will be analyzed later.

**Figure 7:**
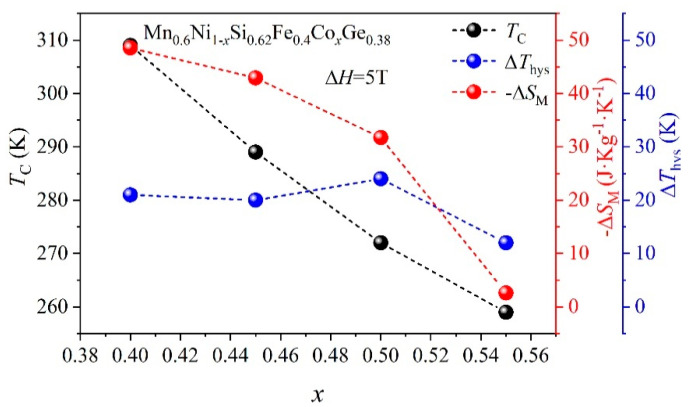
The thermal hysteresis, −Δ*S_M_* and *T_C_* diagrams of HEAs with different Co-doping.

Figure 7 shows the Curie temperature (*T_C_*), isothermal magnetic entropy (Δ*S_M_*), and thermal hysteresis (Δ*T*_hys_) of HEAs with different contents in terms or of Co-doping. The *T_C_*, Δ*S_M_*, and Δ*T*_hys_ of HEAs all decrease gradually with the substitution of Co for Ni. This is the result of a change in the order of magnetic phase transition.

**Figure 8:**
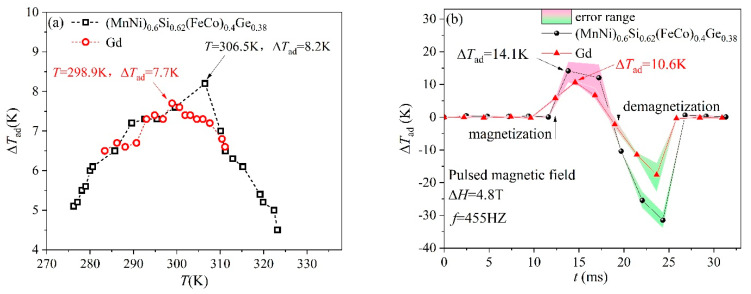
Adiabatic temperature curves of *x* = 0.4 HEA and as a reference Gd under a 5 T magnetic field: (**a**) PPMS superconducting magnetic field; (**b**) 4.8 T pulsed magnetic field.

Adiabatic temperature change (Δ*T_ad_*) is another noteworthy physical property of magnetocaloric alloys. Δ*T_ad_* shows the temperature change when materials enter and leave the magnetic field, measuring the heat transferred from the cold to the hot side of the prototype. In order to obtain the actual Δ*T_ad_* of the material, the *x* = 0.4 HEA was tested using a self-developed PPMS-based adiabatic temperature change direct measurement device. The curves of adiabatic temperature for Ga and *x* = 0.4 HEA, with a superconducting magnetic field at 5 T and a pulsed magnetic field at 4.8 T, are shown in Figure 8a and Figure 8b, respectively. With the gradually increasing temperature, the Δ*T_ad_* of the sample first increased and then decreased, reaching a peak value of 8.2 K at about 306.5 K (very close to the *T_C_* = 309 K). Meanwhile, as a comparison, the maximum Δ*T_ad_* of Gd is 7.7 K at 298.9 K, which is very close to the value reported in the literature [37]. The measured Δ*T_ad_* of Gd and *x* = 0.4 HEA under a 4.8 T pulsed magnetic field are 10.6 K and 14.1 K, respectively. Compared to previous research into magnetocaloric materials, such as HoDyB_2_ [38] with a reversible temperature change of about 1.1 K at 1.93 T and Mn_1.15_Fe_0.8_P_0.5_Si_0.5_C_0.05_ [37] with an adiabatic temperature change of about 3.61 K at 5 T, *x* = 0.4 HEA without rare earth shows a promising magnetocaloric material for application. The applied magnetic field model might lead to different response behaviors for MCE materials, which is consistent with the reported results [39].

In 2018, Law J.Y. et al. [40] reported that the law exponent *n*, among which *n* = 2 is the critical point of the phase transition order, can be calculated through the isothermal magnetic entropy change (Δ*S_M_*) and the applied magnetic field (Δ*H*). A maximum law exponent *n_max_* > 2 indicates that the material belongs to the FOPT material, while *n_max_* < 2 indicates that the material belongs to the (SOPT) material. The calculation formula of law exponent *n* is as follows:

(6) $$n(T,H)=\frac{d\ln\left|∆S_{M}\right|}{d\ln H}$$

**Figure 9:**
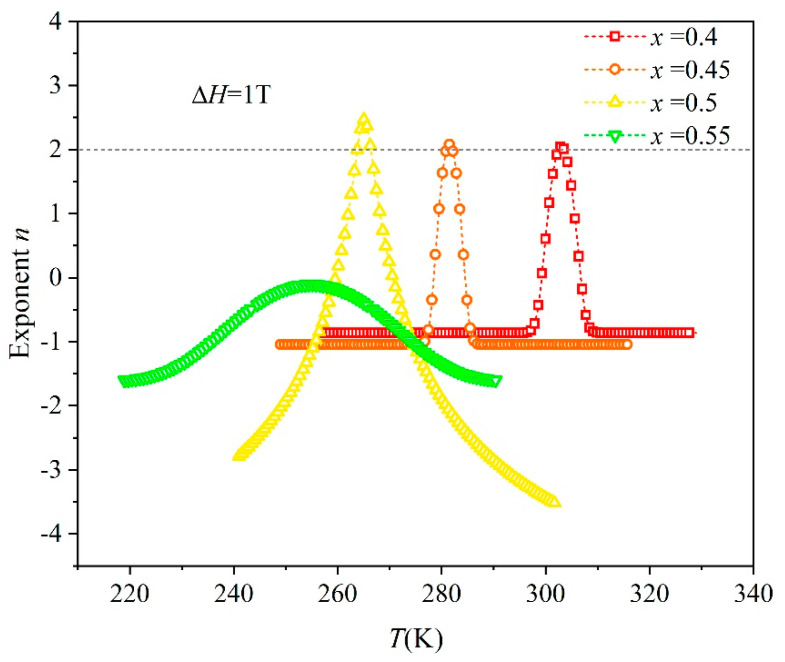
Scatter-line plots of exponent *n* with respect to temperatures for Mn_0.6_Ni_1−*x*_Si_0.62_Fe_0.4_Co*_x_*Ge_0.38_ (*x* = 0.4, 0.45, 0.5, 0.55) alloys.

The law exponent *n* curves obtained after nonlinear fitting of a Gaussian function are shown in Figure 9. For *n_max_* < 2, the alloy of *x* = 0.55 belongs to SOPT materials with very small isothermal magnetic entropy, indicating that it exhibits small MCE. In contrast, the *x* = 0.40, 0.45, and 0.50 alloys for *n_max_* > 2 belong to FOPT materials, which are consistent with the experimental results.

**Figure 10:**
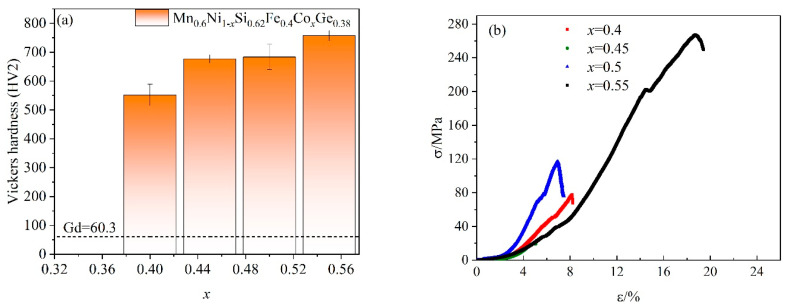
(**a**) Vickers hardness of HEAs with different Co-doping and as a reference gadolinium mental. (**b**) Compressive stress–strain curves of HEAs with different Co-doping.

The Vickers hardness and compression performance of Mn_0.6_Ni_1−*x*_Si_0.62_Fe_0.4_Co*_x_*Ge_0.38_ samples with different Co-doping were investigated, as shown in Figure 10a and Figure 10b, respectively. The Vickers hardness values of the HEAs with different Co-doping are 552 HV2, 677 HV2, 683 HV2, and 758 HV2. The compressive strength of Mn_0.6_Ni_1−*x*_Si_0.62_Fe_0.4_Co*_x_*Ge_0.38_ (*x* = 0.4, 0.45, 0.5, 0.55) alloys are 78 MPa, 21 MPa, 117 MPa, and 267 MPa, as shown in Figure 10b. The reason why the hardness of the alloys increases gradually with the substitution of Co for Ni is related to the order of the phase transition. According to the results obtained from law exponent curves, the *x* = 0.4, 0.45, and 0.5 alloys underwent severe FOPT from hexagonal structures to orthorhombic structures, in which a certain amount of internal stress was generated. In contrast, the *x* = 0.5 alloy exhibited SOPT from paramagnetic phase to ferromagnetic phase without lattice distortion. Therefore, the *x* = 0.5 alloy exhibits better mechanical properties compared to *x* = 0.4, 0.45, and 0.5 alloys.

## 4. Conclusions

In this work, a series of Mn_0.6_Ni_1−*x*_Si_0.62_Fe_0.4_Co*_x_*Ge_0.38_ (*x* = 0.4, 0.45, 0.5, 0.55) HEAs with good magnetocaloric properties are investigated and their structural and magnetic properties are presented. The structure of *x* = 0.45–0.5 alloys is found to be Ni_2_In-type hexagonal with the *P*63/mmc space group near room temperature, while coexisting phase of Ni_2_In-type and TiNiSi-type structure are observed in *x* = 0.4 alloy at 295 K. For *x* = 0.40–0.5, the thermomagnetic phase transition occurs from ferromagnetic martensite to paramagnetic austenite, accompanied by extremely low hysteresis. We demonstrate a remarkable enhancement of isothermal entropy change for 48.5 J∙kg^−1^K^−1^ (*x* = 0.4), comparable to conventional first-order magnetocaloric materials. Thus, the MnNiSi HEA system is promising for room-temperature refrigeration.

## Figures and Tables

**Table 1:** The lattice parameters, phase fraction and phase cell volume of Mn_0.6_Ni_1−*x*_Si_0.62_Fe_0.4_Co*_x_*Ge_0.38_ (*x* = 0.4, 0.45, 0.5, 0.55) alloys at 295 K.

	*x* = 0.4	*x* = 0.45	*x* = 0.5	*x* = 0.55
Ni_2_In-type phase (%)	98.6	100	100	100
TiNiSi-type phase (%)	1.4	0	0	0
a*_hex_*, b*_hex_* (Å)	4.010	4.002	4.000	4.017
c*_hex_* (Å)	5.172	5.161	5.161	5.184
c*_hex_*/a*_hex_*	1.290	1.290	1.290	1.291
a*_orth_* (Å)	6.010	-	-	-
b*_orth_* (Å)	3.645	-	-	-
c*_orth_* (Å)	6.939	-	-	-
a*_orth_*/b*_orth_*	1.649	-	-	-
*v_hex_* (Å^3^)	72.037	71.596	71.525	72.443
*v_orth_* (Å^3^)	152.013	-	-	-
*R_wp-hex_* (%)	5.976	6.138	5.036	3.380
*R_wp-orth_* (%)	3.369	-	-	-

## Data Availability

The original contributions presented in the study are included in the article/supplementary material, further inquiries can be directed to the corresponding author.

## Supplementary Materials

The following supporting information can be downloaded at: https://www.mdpi.com/article/10.3390/e26090799/s1, Figure S1: The EDS mapping of Mn_0.6_Ni_0.5_Si_0.62_Fe_0.4_Co_0.5_Ge_0.38_.
