# Supplementary material for: Enhanced Magnetocaloric Properties of the (MnNi)0.6Si0.62(FeCo)0.4Ge0.38 High-Entropy Alloy Obtained by Co Substitution
Source: Entropy (Basel). 2024 Sep 19;26(9):799. doi: 10.3390/e26090799 (PMC11431282; doi:10.3390/e26090799)
Supplement: Supplementary file 1 [file entropy-26-00799-s001.zip › entropy-3113165-supplementary.pdf]

Enhanced Magnetocaloric Properties of the $(MnNi)_{0.6}Si_{0.62}(FeCo)_{0.4}Ge_{0.38}$ High-Entropy Alloy Obtained by Co Substitution

Zhigang Zheng [1,2,*], Pengyan Huang [1], Xinglin Chen [1], Hongyu Wang [1], Shan Da [1], Gang Wang [1,2], Zhaoguo Qiu [1,2] and Dechang Zeng [1,2]

[1] School of Materials Science & Engineering, South China University of Technology, Guangzhou 510640, China

[2] Yangjiang Branch, Guangdong Laboratory Materials Science and Technology Yangjing Advanced Alloys Laboratory, Yangjiang 529599, China

* Correspondence: mszgzheng@scut.edu.cn

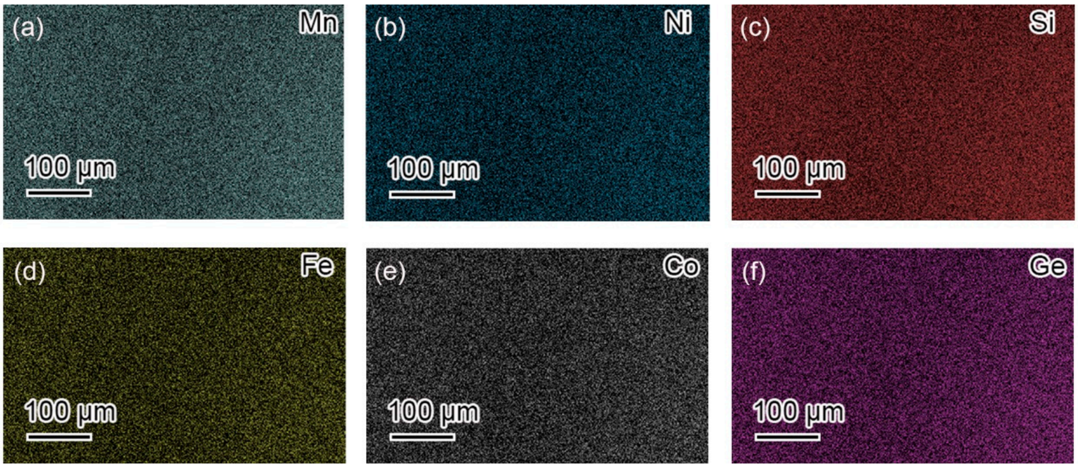

Figure S1. The EDS mapping of $Mn_{0.6}Ni_{0.5}Si_{0.62}Fe_{0.4}Co_{0.5}Ge_{0.38}$
